# Association of serum uric acid levels with COVID-19 severity

**DOI:** 10.1186/s12902-021-00745-2

**Published:** 2021-05-08

**Authors:** Fang Hu, Yifan Guo, Jianghong Lin, Yingjuan Zeng, Juan Wang, Man Li, Li Cong

**Affiliations:** 1 Department of Endocrinology and Metabolism, The Fifth Affiliated Hospital Sun Yat-Sen University, Zhuhai, Guangdong China; 2 Department of Health Management Center, The Fifth Affiliated Hospital Sun Yat-Sen University, Zhuhai, Guangdong China; 3 Key Laboratory of Biomedical Imaging of Guangdong Province, Guangdong Provincial Engineering Research Center of Molecular Imaging, The Fifth Affiliated Hospital Sun Yat-sen University, Zhuhai, Guangdong China; 4 Center for Interventional Medicine, The Fifth Affiliated Hospital Sun Yat-Sen University, Zhuhai, Guangdong China

**Keywords:** COVID-19, Uric acid, Uric acid/creatinine ratio

## Abstract

**Aims:**

Hyperuricemia has attracted increasing attention. However, limited concern has been paid to the potential dangers of lowering serum uric acid (SUA). We observed lower levels of SUA in patients with COVID-19. Therefore, we aim to explore whether patients with COVID-19 had SUA lower than normal and the relationship of SUA and the severity of COVID-19.

**Methods:**

This was a case–control study based on 91 cases with COVID-19 and 273 age- and sex-matched healthy control subjects. We first compared SUA levels and uric acid/creatinine (UA/Cr) ratio between patients with COVID-19 and the healthy controls. Then, we examined the association of SUA levels and UA/Cr ratios with COVID-19 severity in COVID-19 cases only, defined according to the fifth edition of China’s Diagnosis and Treatment Guidelines of COVID-19.

**Results:**

SUA levels in patients with COVID-19 were 2.59% lower, UA/Cr ratios 6.06% lower at admission compared with healthy controls. In sex stratified analysis, levels of SUA and UA/Cr were lower in male patients with COVID-19 while only level of SUA was lower in female patients with COVID-19. Moreover, SUA and UA/Cr values were 4.27 and 8.23% lower in the severe group than that in the moderate group among male COVID-19 patients. Bivariate and partial correlations analysis showed negative correlations between SUA or UA/Cr ratio and COVID-19 after adjusting for age, sex, BMI and eGFR. A multiple linear regression analysis showed that SARS-CoV-2 infection and male sex were independent risk factors associated with lower SUA levels. Male patients with COVID-19 accompanied by low SUA levels had higher risk of developing severe symptoms than those with high SUA levels (incidence rate ratio: 4.05; 95% CI:1.11, 14.72) at admission. Comparing SUA and UA/Cr ratio at three time points (admission, discharge, and follow-up), we found that male patients experienced severe symptoms had lower SUA and UA/Cr ratio levels comparing to moderate patients, but no significant difference between three time points. On the contrary, female patients had lower SUA and UA/Cr ratio at discharge than those at admission, but no significant difference of SUA and UA/Cr ratio between moderate and severe group.

**Conclusion:**

Patients with COVID-19 had SUA and UA/Cr values lower than normal at admission. Male COVID-19 patients with low SUA levels had a significantly higher crude risk of developing severe symptoms than those with high SUA levels. During disease aggravation, the level of SUA gradually decreased until discharge. At the follow-up exam, the level of SUA was similar to the levels at admission.

**Supplementary Information:**

The online version contains supplementary material available at 10.1186/s12902-021-00745-2.

## Introduction

COVID-19 has quickly spread throughout the world. By Mar 8, 2021, there were 116,363,935 cases worldwide and 2587,225 deaths. The mortality rate is as high as 2.22% [1]. However, the pathogenesis of COVID-19 is not clear, and there is currently no effective antiviral treatment. Therefore, it is very important to explore possible treatments according to its pathogenesis.

Although the infection pathways and pathogenesis of different viruses are not the same, the mechanisms by which they cause damage are similar [2]. Viral invasion causes an immune response, induces the activation of inflammatory factors, and causes the production of a large number of free radicals, including ROS (reactive oxygen species) and active nitrogen [3]. These free radicals produce oxidative stress, which can further activate the pathways of inflammatory factors. This cycle could enhance the immune response to eliminate the virus. However, more excessive immune response can also turn the defense mechanism into an injury pathway and aggravate the injury of the body [4]. Thus, oxidative stress plays a crucial role in viral invasion.

Serum uric acid (SUA) is the most abundant antioxidant molecule in the plasma. High SUA levels in humans represent an evolutionary advantage that can enhance antioxidant defense and prolong life [5]. Uric acid (UA) infusion into healthy volunteers increases SUA levels, which is associated with an increase in serum antioxidant capacity [6]. UA restores endothelial function in patients with type 1 diabetes and regular smokers via the antioxidants’ stress response [7]. Therefore, the antioxidant effect of SUA may be potentially beneficial in situations characterized by oxidative stress, although the molecular mechanisms are not fully understood. SUA is thought to have a protective effect on both the central nervous system [8] and primary angle-closure glaucoma [9] against oxidative damage. However, there is a general agreement that hyperuricemia increases the risk of stroke and death [10], cardiovascular diseases [11], gout, insulin resistance, type 2 diabetes [12, 13], and all-cause mortality [14]. The higher mortality associated with more intense reductions in SUA are in line with the U-shaped association of SUA with mortality in some observational studies [15–18]. Hyperuricemia refers to > 420 μmol/L in men and > 360 μmol/L in women. Thus, it may be the most beneficial to control SUA within an appropriate range.

Some studies have investigated the relationship between SUA levels and inflammation (bacteria, viruses, or autoimmunity), but the conclusions were inconsistent [8, 19, 20]. Most studies showed that inflammation could induce the increase of SUA, particularly when the virus invaded the respiratory system [19]. However, SUA tends to decrease during a central nervous system infection [8, 20]. Few studies have examined the association between SUA and COVID-19. SUA levels are clearly elevated in severely ill children compared with non-severely ill children on admission [21]. In our clinical work, we found that the levels of SUA in patients with COVID-19 were lower than average; hence, we aimed to explore the relationship between SUA and COVID-19 to better understand the pathophysiological process of COVID-19.

## Methods

### Data sources

Our hospital, the Fifth Affiliated Hospital Sun Yat-sen University, is the only designated unit for the isolation treatment of COVID-19-diagnosed patients in Zhuhai city, Guangdong province. The study protocol was approved by the ethics committee of Fifth Affiliated Hospital Sun Yat-sen University (SYSU5). We did this study in accordance with the principles of the Declaration of Helsinki and Good Clinical Practice. A total of 364 subjects were studied, including 91 cases (there were 98 cases in our hospital during the period from January 17, 2020 to March 3, 2020, but 6 children were excluded. One patient with a high creatinine level and an estimated glomerular filtration rate (eGFR) of < 60 ml/min*1.73m^2^ was excluded also, and 273 controls from the health management center in our hospital (matched 1:3 with the case group according to gender and age)). Because of the shortage of a healthy population with exact age matches, two 75-year-old female patients were paired with five 75-year-old women and a 74-year-old woman, and a 19-year-old female patient was paired with three 21-year-old female controls. The identification and classification of patients with COVID-19 was based on the criteria of the fifth edition of China’s Diagnosis and Treatment Guidelines of COVID-19 [22]. Patients with COVID-19 were divided into mild, moderate, severe, and critically severe groups (Table S1). Due to a limited sample size, we grouped mild and moderate patients into the moderate group and severe and critically severe patients into the severe group. Nucleic acid tests were performed at Guangdong Center for Disease Control and Prevention. Complete laboratory data were available for both the control group and the case group. We recorded the patients’ sex, age, disease history, laboratory examination, and treatments, with a particular focus on the SUA and creatinine levels at admission, discharge, and follow-up exams. Because renal function has an effect on SUA, we also used SUA/creatinine (UA/Cr) for statistical analysis. The most severe period was indicated by the lowest arterial partial pressure of oxygen (PaO_2_)/fraction of inspiration oxygen (FiO_2_). The Chronic Kidney Disease Epidemiology Collaboration (CKD-EPI) equation was used to calculate eGFR [23].

Fasting blood samples were collected from all patients after admission. Blood leukocyte (LEU), lymphocyte (LYM%), fasting blood glucose (FBG), creatinine, UA, urea, albumin (ALB), globulin (GLB), lactate dehydrogenase (LDH), and a-hydroxybutyrate dehydrogenase (α-HBDH) were obtained in electric medical record system. Antiviral, anti-infective, and supportive treatments were used by the attending doctors according to the patients’ conditions. COVID-19 nucleic acid tests (throat swabs) were performed every other day. Patients whose nasal swabs were negative were considered cured and were discharged from the hospital.

### Statistical analysis

The data were analyzed with SPSS 25.0 (SPSS Inc., Chicago, IL) and R (4.04). Normality was assessed with the Kolmogorov-Smirnoff test. The non-normal data were natural logarithm transformed to a normal distribution. The data that were normally distributed were represented by the mean ± standard deviation (mean ± SD), and the means between two groups were compared using the independent Student’s t-test. Non-normally distributed data were represented by the median and interquartile range [Md (P25–P75)], and the Mann–Whitney U test was used to compare the medians between two groups. Categorical variables were expressed as the frequency (constituent ratio) [n (%)]. The rate or constituent ratio was compared with the Chi-squared test. The linear regression analysis was used to assess the relationships among the SUA levels, the UA/Cr ratio, and COVID-19 outcome. The logistic regression analysis was used to assess the quantitative relationships among the SUA levels, the UA/Cr ratio, and the severity of illness. Incidence rates were calculated as the number of events per person-days. Linear mixed-effects model was used to evaluate the change of the levels of UA and UA/Cr ratio between moderate and severe patients at admission, discharge, and follow-up exams stratified by gender. *P* < 0.05 was considered statistically significant.

## Results

### Characteristics of the study subjects

The age of the COVID-19 group was 47.53 ± 15.43 years old and that of the healthy control group was 47.55 ± 15.33 years old (Table 1). There was no statistically significant difference in age and gender between two groups. The levels of FBG were higher in the case group than those in the controls (*P* < 0.001), whereas the eGFR, HGB, LEU, LYM%, and ALB/GLB values were lower in the case group than those in the controls (Table 1). SUA levels were lower in the COVID-19 group than in the healthy controls (*P* < 0.001 for overall; *P* < 0.001 for males; *P* = 0.001 for females). Serum UA/Cr ratios were also lower in the COVID-19 group than those in the healthy controls (*P* = 0.001 for overall; *P* = 0.002 for males; *P* = 0.07 for females).

**Table 1:** Demographics and clinical characteristics of subjects

Factors	Control group (*n* = 273)	COVID-19		
		Total (*n* = 91)	Moderate group (*n* = 69)	Severe group (*n* = 22)
Age (years)	47.55 ± 15.33	47.53 ± 15.43	44.00 ± 14.32	58.59 ± 13.69^†, ‡^
Gender (male/female)	120/153	40/51	26/43	14/8^‡^
BMI (kg/m^2^)	23.58 ± 3.38	23.57 ± 3.49	23.18 ± 3.41	24.95 ± 3.49^‡^
HGB (g/L)	141.99 ± 13.60	138.23 ± 17.39	138.48 ± 16.61	137.45 ± 20.34
LEU^*^(*10^9^/L)	1.73 ± 0.24	1.59 ± 0.32^†^	1.59 ± 0.32^†^	1.58 ± 0.32^†^
LYM%	35.92 ± 7.90	32.54 ± 10.46^†^	34.17 ± 9.72	27.43 ± 11.27^†, ‡^
eGFR^*^ (ml/min*1.73m^2^)	4.70 ± 0.15	4.66 ± 0.15^†^	4.68 ± 0.15	4.57 ± 0.13^†, ‡^
AST^*^ (U/L)	3.06 ± 0.29	3.03 ± 0.39	2.96 ± 0.36	3.24 ± 0.41^†, ‡^
ALB/GLB	1.74 ± 0.24	1.35 ± 0.22^†^	1.38 ± 0.22^†^	1.24 ± 0.18 ^†, ‡^
α-HBDH^*^ (U/L)	4.91 ± 0.17	4.90 ± 0.23	4.84 ± 0.20^†^	5.06 ± 0.24^†, ‡^
LDH^*^ (U/L)	5.14 ± 0.18	5.14 ± 0.25	5.08 ± 0.22^†^	5.32 ± 0.26^†, ‡^
FPG (mmol/L)	4.66 (4.39–5.05)	5.31 (4.890–6.18) ^†^	5.06 (4.830–5.81) ^†^	5.80 (5.340–7.36) ^†, ‡^
PaO_2_/FiO_2_^*^	–	5.94 ± 0.53	6.19 ± 0.25	5.12 ± 0.36^‡^
Days from onset to admission	–	3.0 (1.0–6.0)	3.0 (1.00–6.0)	4.0 (1.750–6.0)
Days from admission to PCR negative)	–	9.0 (4.00–13.0)	8.0 (4.00–10.0)	11.50 (4.750–20.75) ^‡^
Diabetic patients (%)	0	9 (9.9)	4 (5.8)	5 (22.7) ^‡^
Gout patients (%)	0	0		
Hypertensive patients (%)	0	17 (18.7)	10 (14.5)	7 (31.8)
UA^*^ (μmol/L)				
Total	5.80 ± 0.24	5.65 ± 0.28^†^	5.66 ± 0.29^†^	5.62 ± 0.25^†^
Male	5.95 ± 0.18	5.78 ± 0.26^†^	5.86 ± 0.20^†^	5.61 ± 0.28^†, ‡^
Female	5.68 ± 0.21	5.55 ± 0.26^†^	5.53 ± 0.26^†^	5.63 ± 0.20
Creatinine^*^ (μmol/L)				
Total	4.14 ± 0.20	4.10 ± 0. 26	4.08 ± 0.27	4.16 ± 0.22
Male	4.31 ± 0.13	4.30 ± 0.21	4.32 ± 0.22	4.26 ± 0.19
Female	4.01 ± 0.14	3.94 ± 0.18	3.93 ± 0.19^†^	4.00 ± 0.15
UA/Cr ratio^*^				
Total	1.65 ± 0.20	1.55 ± 0.27^†^	1.58 ± 0.24^†^	1.45 ± 0.32^†^
Male	1.64 ± 0.20	1.48 ± 0.28^†^	1.54 ± 0.23^†^	1.35 ± 0.34^†, ‡^
Female	1.67 ± 0.21	1.60 ± 0.24^†^	1.60 ± 0.25	1.63 ± 0.18

***Abbreviations*:** *BMI* Body mass index, *HGB* Hemoglobin, *LEU* Leukocyte, *LYM* Lymphocyte, *eGFR* Estimated glomerular filtration rate, *AST* Aspartic transaminase, *ALB* Albumin, *GLB* Globulin, *α-HBDH* α-hydroxybutyrate dehydrogenase, *LDH* Lactate dehydrogenase, *FPG* Fasting plasma glucose, *UA* Uric acid, *Cr* Creatinine

***Note***: Data were expressed as the mean ± standard deviation (SD) or median (P25–P75). Days (from onset to admission): the days from symptom appearance to hospitalization, days (from admission to PCR negative): the days from hospitalization to result of nucleic acid of SARS-CoV-2 negative

a*: The data were transformed into Ln (a)

Compared with the control group: *P* < 0.05 labeled as ^†^;

Compared with the moderate group: *P* < 0.05 labeled as ^‡^

COVID-19 patients with severe symptoms were older, more likely to be male, and had a higher BMI, LYM%, eGFR and ALB/GLB.Moreover PaO2/FiO2 was lower in the severe group than that in the moderate group (Table 1). The AST, FBG, α-HBDH, and LDH levels were higher in the severe group than those in the moderate group (Table 1). The number of days from symptom appearance to hospitalization did not differ between the two groups. The days from admission to polymerase chain reaction (PCR) negative was longer in the severe group than that in the moderate group (11.50 (4.750–20.75) vs. 8.0 (4.00–10.0), *P* = 0.04).

### Association of SARS-CoV-2 infection with SUA and UA/Cr

**Fig. 1:**
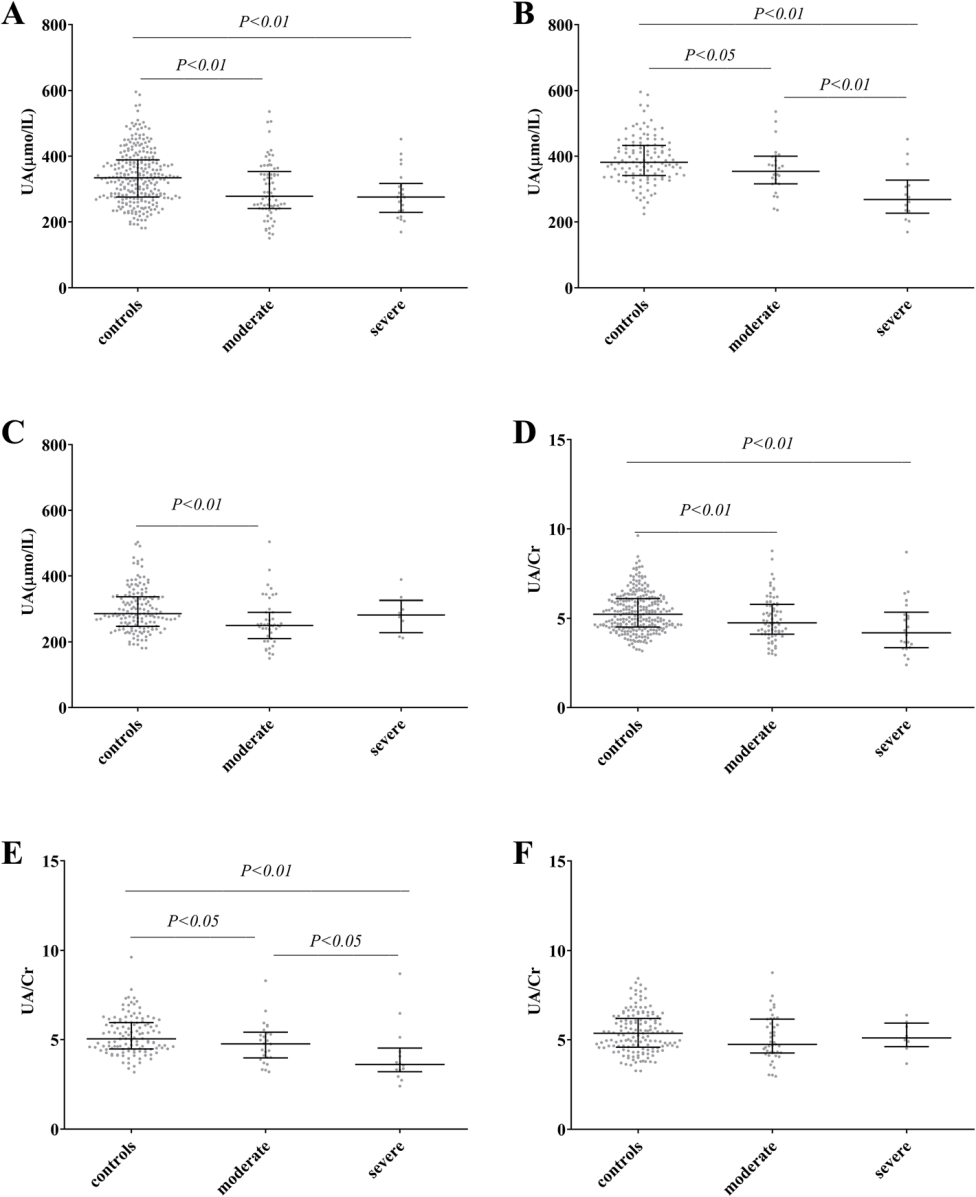
Comparison of SUA and UA/Cr in patients with moderate, severe COVID-19 and control group. Note: Comparison of SUA and UA/Cr in patients with moderate, severe COVID-19 and control group: using Scatter dot plot. The line contained 50% of all values (from 25th to 75th percentile) and was divided by the horizontal bar of the median value (50th percentile). (A) (D) Total subjects. (B)(E) Male subjects (C)(F) Female subjects

Comparing to the age-, sex-matched healthy adults, patients with COVID-19 had lower UA and UA/Cr ratio (Fig. 1a, d) at baseline, despite whether they experienced severe symptoms. Furthermore, specifically in males, the UA level and UA/Cr ratio were lower in severe patients than those in moderate patients (*P* = 0.002 for UA; *P* = 0.046 for UA/Cr ratio; Fig. 1b, e). No statistically significant difference in either UA or UA/Cr ratio was identified in female patients when comparing between severe and moderate groups (Fig. 1c, f). There was a negative correlation between SUA and COVID-19 outcome by either bivariate or partial correlation controlling by age, gender, BMI and eGFR(*p* < 0.001). The same conclusion also could get between UA/Cr and COVID-19 outcome (Table 2). By linear regression analysis, both SARS-CoV-2 infection and male gender were significantly associated with the UA levels after adjusting with other potential confounding factors including LEU, LYM%, eGFR, ALB/GLB and FPG (Table 3). But, SARS-COV-2 infection was not an independent risk factor associated with the UA/Cr levels after adjusting with other potential confounding factors including LEU, LYM%, ALB/GLB and FPG (*P* = 0.07).

**Table 2:** Correlation between COVID-19 and UA^*^, UA/Cr ratio^*^

	Bivariate correlation (*P* value)	partial correlation (*P* value)
UA^*^	**−0.23 (*P*** **< 0.001)**	**− 0.34 (*P*** **< 0.001)**
UA/Cr ratio^*^	**−0.19 (*P*** **< 0.001)**	**− 0.18 (*P*** **= 0.001)**

***Note***: Partial correlation is controlling by age, gender, BMI and eGFR. Spearman test were used

***Abbreviations***: *BMI* Body mass index, *eGFR* Estimated glomerular filtration rate, *UA* Uric acid, *Cr* Creatinine

a^*^: The data were transformed into Ln (a)

**Table 3:** Association of UA^*^, UA/Cr ratio^*^ with COVID-19 and gender

		Model 1	Model 2	Model 3
		*β* (95% CI)	*β* (95% CI)	*β* (95% CI)
UA^#^	COVID-19	−0.25^†^ (− 0. 20, − 0.10)	−0.21^†^ (− 0.20, − 0.06)	−0.15^†^ (− 0.17, − 0.01)
	Male	0.50^†^ (0.22, 0.31)	0.53^†^ (0.22, 0.33)	0.47^†^ (0.19, 0.30)
	COVID-19*Male	–	−0.06 (− 0.15, 0.06)	−0.001 (− 0.10, 0.10)
	AIC	−79.27	−78.03	− 111.46
	BIC	−63.68	−58.54	− 72.49
UA/Cr ratio^#^	COVID-19	−0.21^†^ (− 0.16, − 0.06)	−0.13 (− 0.13, 0.01)	−0.06 (− 0.11, 0.05)
	Male	−0.12^†^ (− 0.10, − 0.01)	−0.07 (− 0.09, 0.02)	−0.08 (− 0.09, − 0.02)
	COVID-19*Male	–	−0.13 (− 0.20, − 0.01)	−0.11 (− 0.18, 0.02)
	AIC	−68.02	−69.28	−83.80
	BIC	− 52.43	−49.80	−48.72

***Note*:** Linear regression analysis was used to assess association of UA*, UA/Cr ratio* with COVID-19 and gender

***Abbreviations*:** *AIC* Akaike information criterion, *BIC* Bayesian Information Criterions

Model 1: Including COVID-19 and gender

Model 2: Including Model 1 and COVID-19*gender

Model 3 of UA^#^: Including Model 2 and LEU^#†^, LYM%^†^, eGFR^#†^, ALB/GLB, FPG^†^

Model 3 of UA/Cr ratio^#^: Including Model 2 and LEU^#†^, LYM%^†^, ALB/GLB, FPG

The *P*-values are for the beta coefficient

a^#^: The data were transformed into Ln (a)

^†^*P* <0.05

### Association with COVID-19 severity

We grouped population to High-UA and Low-UA according to the levels of SUA median and gender. In male patients with COVID-19, the incidence rate of developing severe symptoms was 4.05-fold higher (95% CI: 1.11, 14.72) in low-UA group compared to the high-UA group. Nevertheless, there is no statistically significant difference in the incidence rate of developing severe symptoms when comparing between the low versus high-UA group in the female strata (incidence rate ratio 0.26; 95% CI: 0.05, 1.29; Table 4).

**Table 4:** Incidence rate ratio of COVID-19 patients

	UA Group	Severe COVID-19	Person-Days	Incidence Rate	Incidence Rate Ratio (95% CI)
Total	High-UA (*n* = 45)	10 (22.2%)	323	0.031	0.84 (0.36, 1.98)
	Low-UA (n = 45)	11 (24.4%)	423	0.026	
Male	High-UA (*n* = 19)	3 (15.8%)	152	0.020	4.05 (1.11, 14.72)
	Low-UA (*n* = 20)	10 (55.0%)	123	0.081	
Female	High-UA (*n* = 25)	6 (24.0%)	257	0.023	0.26 (0.05, 1.29)
	Low-UA (*n* = 26)	2 (7.7%)	313	0.006	

***Notes*:** the cut-off point of total patients between high-UA and low-UA is 277 μmol /L (Median)

the cut-off point of male patients between high-UA and low-UA is 334 μmol /L (Median)

the cut-off point of female patients between high-UA and low-UA is 252 μmol /L (Median)

Days referred to the time from admission to the severe period

One patient was not analyzed because he was severe type at admission

Then, we assessed the correlation between PaO2/FiO2 and UA, UA/Cr ratio in male patients of COVID-19 by Pearson test. PaO2/FiO2, as an important respiratory parameter, could roughly reflect the severity of illness. As shown in Table 5, there was positive correlation between SUA and PaO2/FiO2 by either bivariate or partial correlation controlling by age, BMI and eGFR(*p* < 0.05). But there was no association between UA/Cr and PaO2/FiO2 by partial correlation controlling by age, BMI and eGFR (*p* = 0.058).

**Table 5:** Correlation between PaO_2_/FiO_2_^*^ and UA^*^, UA/Cr ratio^*^ in male patients of COVID-19

	Bivariate correlation (*P* value)	partial correlation (*P* value)
UA^*^	**0.463 (*P*** **= 0.003)**	**0.411 (*P*** **= 0.013)**
UA/Cr ratio^*^	**0.361 (*P*** **= 0.024)**	**0.319 (P = 0.058)**

***Note***: Partial correlation is controlling by age, BMI and eGFR. Pearson test were used

***Abbreviations***: *BMI* Body mass index, *eGFR* Estimated glomerular filtration rate, *UA* Uric acid, *Cr* creatinine

a^*^: The data were transformed into Ln (a)

As shown in Table 6, the univariate analysis revealed that both UA and UA/Cr were associated with the severity of COVID-19 (OR 0.01; 95% CI: 0.00, 0.30; *P* = 0.008 for UA OR 0.07; 95% CI: 0.004, 1.07; *P* = 0.01 for UA/CR). However, these associations did not remain statistically significant after adjusting for other potential confounding factors. SUA and UA/Cr on admission were not independent risk factors for the severity of COVID-19.

**Table 6:** Linear mixed effects models of changes in the levels of UA and UA/Cr ratio between severe and moderate symptoms and three time points by gender

Fixed Effects	UA				UA/Cr ratio			
	Male		Female		Male		Female	
	Effect Size	95% CI	Effect Size	95% CI	Effect Size	95% CI	Effect Size	95% CI
Moderate	Ref	Ref	Ref	Ref	Ref	Ref	Ref	Ref
Severe	**− 0.17**	**(− 0.29, − 0.05)**	0.07	(− 0.11, 0.24)	**− 0.19**	**(− 0.35, − 0.04)**	0.02	(− 0.15, 0.19)
Admission	Ref	Ref	Ref	Ref	Ref	Ref	Ref	Ref
Discharge	− 0.05	(− 0.12, 0.01)	**− 0.11**	**(− 0.18, − 0.04)**	−0.06	(− 0.14, 0.01)	**− 0.18**	**(− 0.25, − 0.12)**
Follow-up	0.04	(− 0.04, 0.13)	0.04	(− 0.03, 0.11)	0.05	(− 0.04, 0.14)	−0.03	(− 0.10, 0.04)

***Abbreviations*:** *UA* Uric acid, *Cr* Creatinine

### Longitudinal effect of the virus on SUA

**Fig. 2:**
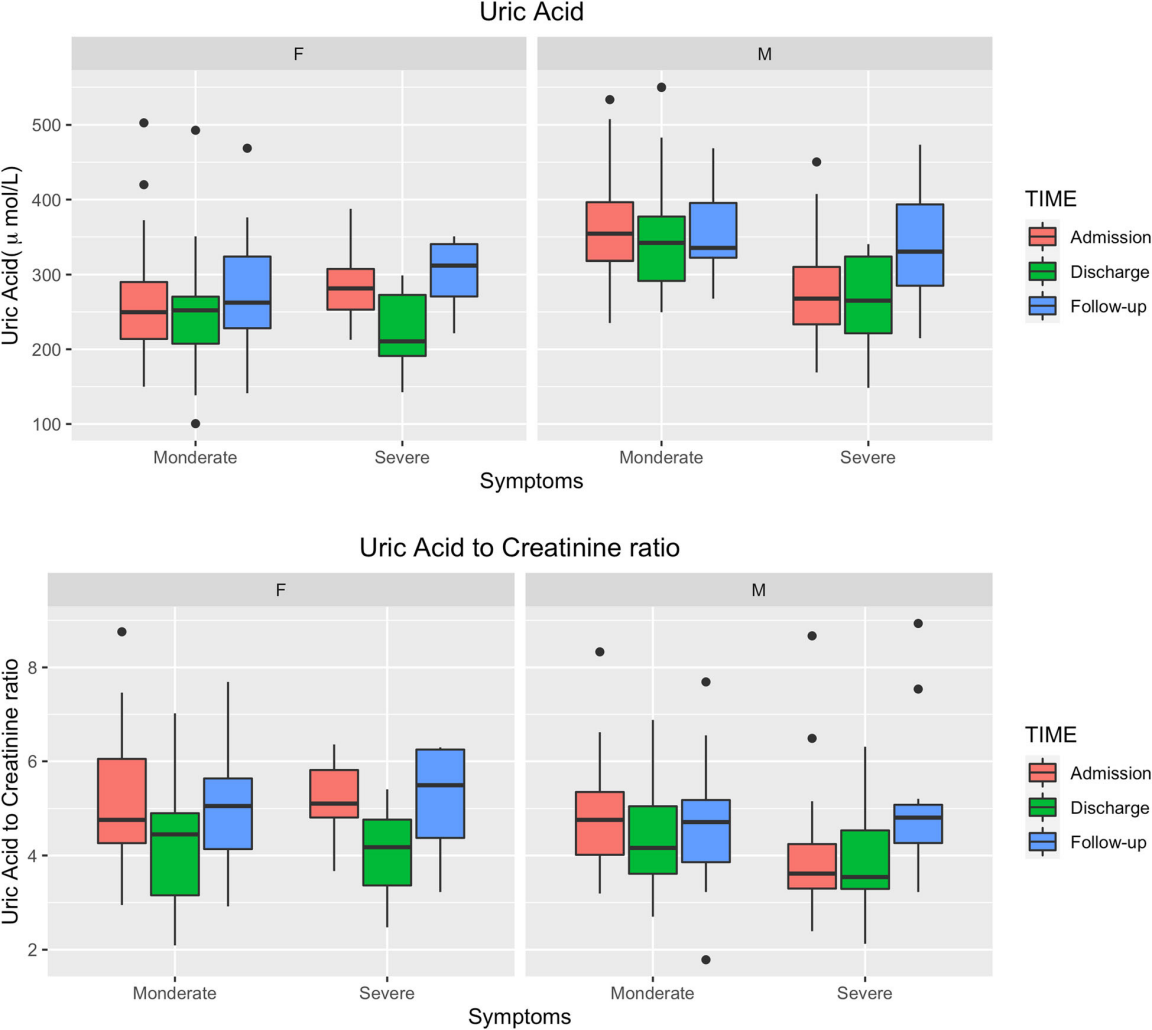
Boxplots of the levels SUA and UA/Cr ratio by gender and symptom levels at admission (*N* = 91), discharge (*N* = 90), and follow-up time (*N* = 68). Note: Comparison of SUA and UA/Cr in patients with moderate and severe COVID-19 among three time points (admission, discharge and follow-up). The line contained 50% of all values (from 25th to 75th percentile) and was divided by the horizontal bar of the median value (50th percentile). Abbreviations: F:female; M: male

We fitted linear mixed-effects models with random intercept and slope for time to investigate whether changes in UA and UA/Cr ratio over time were associated with the severity level of COVID-19 symptoms when stratified by gender. In males, patients with severe symptoms had significantly lower SUA and UACR levels comparing to moderate patients (SUA effect size − 0.17, 95% CI -0.29, − 0.05; UACR effect size − 0.19, 95% CI -0.35, − 0.04), however we did not observe significant difference between different time points (Table 7). In females, we found no statistical difference of either SUA or UACR levels between severe patients and moderate patients. Nevertheless, female patients have lower SUA and UACR levels at discharge comparing to their levels at admission (SUA effect size − 0.11, 95% CI -0.18, − 0.04; UACR effect size − 0.18, 95% CI -0.25, − 0.12). At first follow-up exam, these differences disappeared (Fig. 2).

**Table 7:** Levels of UA and UA/Cr ratio between severe and moderate symptoms and three time points by gender

Fixed Effects	UA				UA/Cr ratio			
	Male		Female		Male		Female	
	Effect Size	95% CI	Effect Size	95% CI	Effect Size	95% CI	Effect Size	95% CI
Moderate	Ref	Ref	Ref	Ref	Ref	Ref	Ref	Ref
Severe	**−0.17**	**(− 0.29, − 0.05)**	0.07	(−0.11, 0.24)	**− 0.19**	**(− 0.35, − 0.04)**	0.02	(−0.15, 0.19)
Admission	Ref	Ref	Ref	Ref	Ref	Ref	Ref	Ref
Discharge	−0.05	(−0.12, 0.01)	**−0.11**	**(− 0.18, − 0.04)**	−0.06	(− 0.14, 0.01)	**−0.18**	**(− 0.25, − 0.12)**
Follow-up	0.04	(− 0.04, 0.13)	0.04	(− 0.03, 0.11)	0.05	(− 0.04, 0.14)	−0.03	(− 0.10, 0.04)

***Note***: Linear mixed effects models were used to assess changes of levels of UA and UA/Cr ratio between severe and moderate symptoms and three time points by gender

***Abbreviations***: *UA* Uric acid, *Cr* Creatinine

## Discussion

Our study showed that SUA and UA/Cr levels at admission were lower in patients with COVID-19 than controls, particularly in males. Moreover, SUA and UA/Cr values were lower in the severe group than in the moderate group among male patients with COVID-19. Male patients with COVID-19 with low SUA levels at had a higher risk of developing severe symptoms than those with high SUA levels. There was a positive correlation between SUA and PaO_2_/FiO_2_. During disease aggravation, the level of SUA gradually decreased until discharge. Hence, SUA is closely related to the severity of COVID-19, although it is not an independent risk factor, regardless of whether low SUA is the cause or result of the illness.

SUA is a powerful antioxidant that accounts for over half of the free radical scavenging activity in human blood by reducing superoxide and singlet oxygen and protecting the oxidation of vitamin C through the chelation of iron [24]. Since neurons are highly susceptible to oxidative stress, decreased SUA levels are present in central nervous system disorders such as Alzheimer’s disease [25], Guillain-Barre syndrome and many types of meningitis (viral meningitis or meningoencephalitis, brain cysticercosis, tuberculous meningitis or meningoencephalitis, cryptococcus meningitis or meningoencephalitis, and bacterial meningitis or meningoencephalitis) [26]. Conversely, SUA levels are increased in infections of other systems. Respiratory syncytial virus (RSV) induces increased UA levels in mouse neonates, and the inhibition of UA by xanthine oxidase inhibitor decreases mucus production, reduces cellular infiltrates to the lungs (particularly ILC2s), and decreases type 2 immune responses [19]. UA is a biomarker of early cystic fibrosis lung disease [27], and high SUA is positively correlated with severe infections such as sepsis [28]. Hence, it is important to monitor changes of SUA level in infectious diseases.

In our clinical observation, patients with COVID-19 infection had lower SUA levels than the normal range. It is consistent with previous research findings [29, 30]. Severely infected patients had lower SUA levels, and this trend was more obvious in men. However, the mechanism was unclear. First, as a primary antioxidant, SUA could be consumed by oxidizing agents to prevent an inflammatory response. Therefore, systemic inflammation and oxidative stress were likely to cause an obvious consumption of UA and a significant decrease in its serum levels. We suggested that the decreased SUA levels may play a part in the anti-oxidative insufficiency, which could contribute to COVID-19 development. Second, serum metabolomic analysis of patients with COVID-19 showed that guanosine monophosphate (GMP) levels were lower in patients with COVID-19 than in healthy people. In addition, GMP levels were lower in the severe group compared with the moderate group [31]. GMP is eventually metabolized into SUA and excreted out of the body. Thus, SUA decreases following decreased GMP levels in patients with COVID-19. Finally, CD39 and CD73 increase because of inflammation. Increased CD39 and CD73 could break adenosine triphosphate (ATP) down into adenosine monophosphate (AMP) and AMP down into adenosine. Hence, we speculated AMP was decreased in patients with COVID-19. While AMP is a raw material of SUA, SUA was reduced in patients with COVID-19 following [32]. Finally, low SUA might occur due to a specific dysfunction of the kidney proximal tubule caused by COVID-19 [30].

We found that patients with low SUA and UA/Cr levels at admission had a higher incidence rate of developing severe symptoms of COVID-19 later. However, SUA levels and UA/Cr were not the independent risk factors of developing severe disease. Thus, the deterioration of the disease may be the result of the joint action of multiple factors. It is known that hyperuricemia increases the risk of stroke and death, cardiovascular diseases, and gout. Moreover, hypouricemia is now recognized to increase adverse disease outcome; however, the cutoff is difficult to determine. Determining the roles of SUA and oxidative stress in COVID-19 is quite difficult. The mechanism of SUA in the pathogenesis of COVID-19 is should be further explored in a future study with a larger sample size. Increasing SUA levels may be a potential COVID-19 treatment method.

Our study also noted that the relationship between SUA, UA/Cr, and COVID-19 was more obvious in the male population. There were no relevant previous studies reporting this finding. SUA levels were higher in men than in women among healthy people. Testosterone might upregulate the expression of the urater transporter 1 gene, thereby increasing the reabsorption of UA and the level of SUA [33]. In addition, SUA had different effects on the incidence of thyroid nodules [34] and fat distribution [35] in different genders. We speculated that the male oxidative stress response was stronger than the female response, so more SUA must be consumed in males. Thus, SUA might play a more important role in oxidative stress in males.

In addition, previous research showed that SUA levels were clearly elevated in severely ill children compared with non-severely ill children on admission. Our study population was adults; hence, the conclusion was opposite. Confirmation of changes in SUA levels with infectious disease in different age groups requires a future study with a larger sample size.

Our data also showed that although patients in both the severe and moderate groups had met the hospital discharge criteria in which they were required to have two consecutive negative COVID-19 nucleic acid tests, their SUA levels deceased upon discharge in female patients, which suggests that these discharged patients had not fully recovered physiologically from the impacts of COVID-19. These patients require further strengthening, nutritional support, and rest. In addition, we found that SUA and UA/Cr played the same role in predicting the severity of the disease in patients with COVID-19 with a normal level of creatinine.

We acknowledge that our present study has some limitations. First, the patients with COVID-19 were divided into mild, moderate, severe, and most severe according to the fifth edition of China’s Diagnosis and Treatment Guidelines of COVID-19. We grouped the mild and moderate patients in the moderate group and the severe and most severe patients in the severe group because of the limited number of patients. Second, it was uncertain whether low SUA levels can contribute to a higher risk of COVID-19 infection because of the lack of SUA level data prior to admission. Third, the relationship between the change of SUA and the risk to severe disease was uncertain because of the lack of regular SUA examinations.

The present study demonstrated that SUA levels and the UA/Cr ratio were decreased and negatively associated with COVID-19 severity, which suggests a possible association between SUA levels with the development of COVID-19 and the involvement of oxidative stress in the pathogenesis of COVID-19.

### Supplementary Information

**Additional file 1: Table 1S** Clinical classification of the COVID-19.

## Data Availability

All data generated or analyzed during this study are included in this published article [and its supplementary information files].

## Appendix

### Statement of guideline

**Table 8:** STROBE Statement—Checklist of items that should be included in reports of case-control studies

	Item No	Recommendation	Page No
**Title and abstract**	1	(*a*) Indicate the study’s design with a commonly used term in the title or the abstract	1
		(*b*) Provide in the abstract an informative and balanced summary of what was done and what was found	5–7
**Introduction**			
Background/rationale	2	Explain the scientific background and rationale for the investigation being reported	8–9
Objectives	3	State specific objectives, including any prespecified hypotheses	9–10
**Methods**			
Study design	4	Present key elements of study design early in the paper	10–12
Setting	5	Describe the setting, locations, and relevant dates, including periods of recruitment, exposure, follow-up, and data collection	10–12
Participants	6	(*a*) Give the eligibility criteria, and the sources and methods of case ascertainment and control selection. Give the rationale for the choice of cases and controls	10–11
		(*b*) For matched studies, give matching criteria and the number of controls per case	10–11
Variables	7	Clearly define all outcomes, exposures, predictors, potential confounders, and effect modifiers. Give diagnostic criteria, if applicable	10–12
Data sources/ measurement	8*	For each variable of interest, give sources of data and details of methods of assessment (measurement). Describe comparability of assessment methods if there is more than one group	10–12
Bias	9	Describe any efforts to address potential sources of bias	10–12
Study size	10	Explain how the study size was arrived at	10–11
Quantitative variables	11	Explain how quantitative variables were handled in the analyses. If applicable, describe which groupings were chosen and why	11–12
Statistical methods	12	(*a*) Describe all statistical methods, including those used to control for confounding	12–13
		(*b*) Describe any methods used to examine subgroups and interactions	12–13
		(*c*) Explain how missing data were addressed	10
		(*d*) If applicable, explain how matching of cases and controls was addressed	10–11
		(*e*) Describe any sensitivity analyses	12–13
**Results**			
Participants	13*	(a) Report numbers of individuals at each stage of study—eg numbers potentially eligible, examined for eligibility, confirmed eligible, included in the study, completing follow-up, and analysed	13–14
		(b) Give reasons for non-participation at each stage	–
		(c) Consider use of a flow diagram	14
Descriptive data	14*	(a) Give characteristics of study participants (eg demographic, clinical, social) and information on exposures and potential confounders	13–14
		(b) Indicate number of participants with missing data for each variable of interest	–
Outcome data	15*	Report numbers in each exposure category, or summary measures of exposure	14–17
Main results	16	(*a*) Give unadjusted estimates and, if applicable, confounder-adjusted estimates and their precision (eg, 95% confidence interval). Make clear which confounders were adjusted for and why they were included	14–17
		(*b*) Report category boundaries when continuous variables were categorized	14–17
		(*c*) If relevant, consider translating estimates of relative risk into absolute risk for a meaningful time period	14–17
Other analyses	17	Report other analyses done—eg analyses of subgroups and interactions, and sensitivity analyses	–
**Discussion**			
Key results	18	Summarise key results with reference to study objectives	18–21
Limitations	19	Discuss limitations of the study, taking into account sources of potential bias or imprecision. Discuss both direction and magnitude of any potential bias	22–23
Interpretation	20	Give a cautious overall interpretation of results considering objectives, limitations, multiplicity of analyses, results from similar studies, and other relevant evidence	21–22
Generalisability	21	Discuss the generalisability (external validity) of the study results	21–22
**Other information**			
Funding	22	Give the source of funding and the role of the funders for the present study and, if applicable, for the original study on which the present article is based	23

## Abbreviations

SUA: Serum uric acid
COVID-19: Coronavirus disease
Cr: creatinine
SARS-CoV-2: Severe acute respiratory syndrome coronavirus 2
ROS: Reactive oxygen species
UA: Uric acid
SYSU5: Fifth Affiliated Hospital Sun Yat-sen University
eGFR: Estimated glomerular filtration rate
PaO_2_: Partial pressure of oxygen
FiO_2_: Fraction of inspiration oxygen
CKD-EPI: Chronic kidney disease epidemiology collaboration
LEU: Leukocyte
NEU: Neutrophil
FBG: Fasting blood glucose
α-HBDH: α-hydroxybutyrate dehydrogenase
HGB: Hemoglobin
LYM: Lymphocyte
BMI: Body mass index
PCR: Polymerase chain reaction
RSV: Respiratory syncytial virus
GMP: Guanosine monophosphate
ATP: Adenosine triphosphate
AMP: Adenosine monophosphate
