# Supplementary material for: Association of serum uric acid levels with COVID-19 severity
Source: BMC Endocr Disord. 2021 May 8;21:97. doi: 10.1186/s12902-021-00745-2 (PMC8106517; doi:10.1186/s12902-021-00745-2)
Supplement: Supplementary file 1 — Additional file 1: Table 1S Clinical classification of the COVID-19. [file 12902_2021_745_MOESM1_ESM.zip › COVID-19 guideline_ESM.pdf]

新型冠状病毒感染的肺炎诊疗方案

（试行第五版）

2019年12月以来，湖北省武汉市陆续发现了多例新型冠状病毒感染的肺炎患者，随着疫情的蔓延，我国其他地区及境外也相继发现了此类病例。该病作为急性呼吸道传染病已纳入《中华人民共和国传染病防治法》规定的乙类传染病，按甲类传染病管理。

随着疾病认识的深入和诊疗经验的积累，我们对《新型冠状病毒感染的肺炎诊疗方案（试行第四版）》进行修订，形成了《新型冠状病毒感染的肺炎诊疗方案（试行第五版）》。

一、病原学特点

新型冠状病毒属于β属的冠状病毒，有包膜，颗粒呈圆形或椭圆形，常为多形性，直径60-140nm。其基因特征与SARSr-CoV和MERSr-CoV有明显区别。目前研究显示与蝙蝠SARS样冠状病毒（bat-SL-CoVZC45）同源性达85%以上。体外分离培养时，2019-nCoV 96个小时左右即可在人呼吸道上皮细胞内发现，而在Vero E6和Huh-7细胞系中分离培养需约6天。

对冠状病毒理化特性的认识多来自对SARS-CoV和MERS-CoV的研究。病毒对紫外线和热敏感，56℃ 30分钟、乙醚、75%乙醇、含氯消毒剂、过氧乙酸和氯仿等脂溶剂均可有效灭活病毒，氯己定不能有效灭活病毒。

二、流行病学特点

（一）传染源。

目前所见传染源主要是新型冠状病毒感染的患者。无症状感染者也可能成为传染源。

（二）传播途径。

经呼吸道飞沫和接触传播是主要的传播途径。气溶胶和消化道等传播途径尚待明确。

（三）易感人群。

人群普遍易感。

三、临床特点

（一）临床表现。

基于目前的流行病学调查，潜伏期1-14天，多为3-7天。

以发热、乏力、干咳为主要表现。少数患者伴有鼻塞、流涕、咽痛和腹泻等症状。重症患者多在发病一周后出现呼吸困难和/或低氧血症，严重者快速进展为急性呼吸窘迫综合征、脓毒症休克、难以纠正的代谢性酸中毒和出凝血功能障碍等。值得注意的是重型、危重型患者病程中可为中低热，甚至无明显发热。

轻型患者仅表现为低热、轻微乏力等，无肺炎表现。

从目前收治的病例情况看，多数患者预后良好，少数患者病情危重。老年人和有慢性基础疾病者预后较差。儿童病例症状相对较轻。

（二）实验室检查。

发病早期外周血白细胞总数正常或减少，淋巴细胞计数减少，部分患者可出现肝酶、乳酸脱氢酶（LDH）、肌酶和肌红蛋白增高；部分危重者可见肌钙蛋白增高。多数患者C反应蛋白（CRP）和血沉升高，降钙素原正常。严重者D-二聚体升高、外周血淋巴细胞进行性减少。

在鼻咽拭子、痰、下呼吸道分泌物、血液、粪便等标本中可检测出新型冠状病毒核酸。

（三）胸部影像学。

早期呈现多发小斑片影及间质改变，以肺外带明显。进而发展为双肺多发磨玻璃影、浸润影，严重者可出现肺实变，胸腔积液少见。

四、诊断标准

湖北以外省份：

（一）疑似病例。

结合下述流行病学史和临床表现综合分析：

1.流行病学史

（1）发病前14天内有武汉市及周边地区，或其他有病例报告社区的旅行史或居住史；

（2）发病前14天内与新型冠状病毒感染者（核酸检测阳性者）有接触史；

（3）发病前14天内曾接触过来自武汉市及周边地区，或来自有病例报告社区的发热或有呼吸道症状的患者；

（4）聚集性发病。

2.临床表现

（1）发热和/或呼吸道症状;

（2）具有上述肺炎影像学特征;

（3）发病早期白细胞总数正常或降低，或淋巴细胞计数减少。

有流行病学史中的任何一条，且符合临床表现中任意2条。无明确流行病学史的，符合临床表现中的3条。

（二）确诊病例。

疑似病例，具备以下病原学证据之一者:

1.呼吸道标本或血液标本实时荧光RT-PCR检测新型冠状病毒核酸阳性;

2.呼吸道标本或血液标本病毒基因测序，与已知的新型冠状病毒高度同源。

湖北省:

（一）疑似病例。

结合下述流行病学史和临床表现综合分析:

1.流行病学史

（1）发病前14天内有武汉市及周边地区，或其他有病例报告社区的旅行史或居住史;

（2）发病前14天内与新型冠状病毒感染者（核酸检测阳性者）有接触史;

（3）发病前14天内曾接触过来自武汉市及周边地区，或来自有病例报告社区的发热或有呼吸道症状的患者;

（4）聚集性发病。

2.临床表现

（1）发热和/或呼吸道症状；

（2）发病早期白细胞总数正常或减少，或淋巴细胞计数减少。

有流行病学史中的任何一条或无流行病学史，且同时符合临床表现中2条。

（二）临床诊断病例。

疑似病例具有肺炎影像学特征者。

（三）确诊病例。

临床诊断病例或疑似病例，具备以下病原学证据之一者：

1.呼吸道标本或血液标本实时荧光RT-PCR检测新型冠状病毒核酸阳性；

2.呼吸道标本或血液标本病毒基因测序，与已知的新型冠状病毒高度同源。

五、临床分型

（一）轻型。

临床症状轻微，影像学未见肺炎表现。

（二）普通型。

具有发热、呼吸道等症状，影像学可见肺炎表现。

（三）重型。

符合下列任何一条：

1.呼吸窘迫，RR≥30次/分；

2.静息状态下，指氧饱和度≤93%；

3.动脉血氧分压（PaO2）/吸氧浓度（FiO2）≤300mmHg

（1mmHg=0.133kPa）。

（四）危重型。

符合以下情况之一者：

1.出现呼吸衰竭，且需要机械通气；

2.出现休克；

3.合并其他器官功能衰竭需ICU监护治疗。

六、鉴别诊断

主要与流感病毒、副流感病毒、腺病毒、呼吸道合胞病毒、鼻病毒、人偏肺病毒、SARS冠状病毒等其他已知病毒性肺炎鉴别，与肺炎支原体、衣原体肺炎及细菌性肺炎等鉴别。此外，还要与非感染性疾病，如血管炎、皮肌炎和机化性肺炎等鉴别。

七、病例的发现与报告

湖北以外省份：

各级各类医疗机构的医务人员发现符合病例定义的疑似病例后，应当立即进行隔离治疗，院内专家会诊或主诊医师会诊，仍考虑疑似病例，在2小时内进行网络直报，并采集标本进行新型冠状病毒核酸检测，同时在确保转运安全前提下立即将疑似患者转运至定点医院。与新型冠状病毒感染者有密切接触的患者，即便常见呼吸道病原检测阳性，也建议及时进行新型冠状病毒病原学检测。

疑似病例连续两次呼吸道病原核酸检测阴性（采样时间至少间隔1天），方可排除。

湖北省：

各级各类医疗机构的医务人员发现符合病例定义的疑似病和临床诊断病例后，应当立即进行隔离治疗，疑似病例和临床诊断病例要单间隔离，对疑似病例和临床诊断病例要尽快采集标本进行病原学检测。

八、治疗

（一）根据病情确定治疗场所。

1. 疑似及确诊病例应当在具备有效隔离条件和防护条件的定点医院隔离治疗，疑似病例应当单人单间隔离治疗，确诊病例可多人收治在同一病室。

2. 危重型病例应当尽早收入 ICU 治疗。

（二）一般治疗。

1. 卧床休息，加强支持治疗，保证充分热量；注意水、电解质平衡，维持内环境稳定；密切监测生命体征、指氧饱和度等。

2. 根据病情监测血常规、尿常规、CRP、生化指标（肝酶、心肌酶、肾功能等）、凝血功能、动脉血气分析、胸部影像学等。有条件者可行细胞因子检测。

3. 及时给予有效氧疗措施，包括鼻导管、面罩给氧和经鼻高流量氧疗。

4. 抗病毒治疗：目前没有确认有效的抗病毒治疗方法。可试用α-干扰素雾化吸入（成人每次 500 万 U 或相当剂量，加入灭菌注射用水 2ml，每日 2 次）、洛匹那韦/利托那韦（200 mg/50 mg，每粒）每次 2 粒，每日 2 次，或可加用利巴韦林（成人首剂 4g，次日每 8 小时一次，每次 1.2g，或 8mg/kg iv.

每 8 小时一次）。要注意洛匹那韦/利托那韦相关腹泻、恶心、呕吐、肝功能损害等不良反应，同时要注意和其他药物的相互作用。

5.抗菌药物治疗：避免盲目或不恰当使用抗菌药物，尤其是联合使用广谱抗菌药物。

（三）重型、危重型病例的治疗。

1.治疗原则：在对症治疗的基础上，积极防治并发症，治疗基础疾病，预防继发感染，及时进行器官功能支持。

2.呼吸支持：

(1)氧疗：重型患者应当接受鼻导管或面罩吸氧，并及时评估呼吸窘迫和（或）低氧血症是否缓解。

(2)高流量鼻导管氧疗或无创机械通气：当患者接受标准氧疗后呼吸窘迫和（或）低氧血症无法缓解时，可考虑使用高流量鼻导管氧疗或无创通气。若短时间（1-2 小时）内病情无改善甚至恶化，应当及时进行气管插管和有创机械通气。

(3)有创机械通气：采用肺保护性通气策略，即小潮气量（4-8ml/kg 理想体重）和低吸气压力（平台压<30cmH2O）进行机械通气，以减少呼吸机相关肺损伤。较多患者存在人机不同步，应当及时使用镇静以及肌松剂。

(4)挽救治疗：对于严重 ARDS 患者，建议进行肺复张。在人力资源充足的情况下，每天应当进行 12 小时以上的俯卧位通气。俯卧位通气效果不佳者，如条件允许，应当尽快考虑体外膜肺氧合（ECMO）。

3.循环支持：充分液体复苏的基础上，改善微循环，使用血管活性药物，必要时进行血流动力学监测。

4.其他治疗措施

可根据患者呼吸困难程度、胸部影像学进展情况，酌情短期内（3～5日）使用糖皮质激素，建议剂量不超过相当于甲泼尼龙1～2mg/kg/日，应当注意较大剂量糖皮质激素由于免疫抑制作用，会延缓对冠状病毒的清除；可静脉给予血必净100ml/次，每日2次治疗；可使用肠道微生态调节剂，维持肠道微生态平衡，预防继发细菌感染；可采用恢复期血浆治疗；对有高炎症反应的危重患者，有条件可以考虑使用体外血液净化技术。

患者常存在焦虑恐惧情绪，应当加强心理疏导。

（四）中医治疗。

本病属于中医疫病范畴，病因为感受疫戾之气，各地可根据病情、当地气候特点以及不同体质等情况，参照下列方案进行辨证论治。

1.医学观察期

临床表现1：乏力伴胃肠不适

推荐中成药：藿香正气胶囊（丸、水、口服液）

临床表现2：乏力伴发热

推荐中成药：金花清感颗粒、连花清瘟胶囊（颗粒）、疏风解毒胶囊（颗粒）、防风通圣丸（颗粒）

2.临床治疗期

（1）初期：寒湿郁肺

临床表现：恶寒发热或无热，干咳，咽干，倦怠乏力，胸闷，脘痞，或呕恶，便溏。舌质淡或淡红，苔白腻，脉濡。

推荐处方：苍术 15g、陈皮 10g、厚朴 10g、藿香 10g、草果 6g、生麻黄 6g、羌活 10g、生姜 10g、槟郎 10g

（2）中期：疫毒闭肺

临床表现：身热不退或往来寒热，咳嗽痰少，或有黄痰，腹胀便秘。胸闷气促，咳嗽喘憋，动则气喘。舌质红，苔黄腻或黄燥，脉滑数。

推荐处方：杏仁 10g、生石膏 30g、瓜蒌 30g、生大黄 6g（后下）、生炙麻黄各 6g、葶苈子 10g、桃仁 10g、草果 6g、槟郎 10g、苍术 10g

推荐中成药：喜炎平注射剂，血必净注射剂

（3）重症期：内闭外脱

临床表现：呼吸困难、动辄气喘或需要辅助通气，伴神昏，烦躁，汗出肢冷，舌质紫暗，苔厚腻或燥，脉浮大无根。

推荐处方：人参 15g、黑顺片 10g（先煎）、山茱萸 15g，送服苏合香丸或安宫牛黄丸

推荐中成药：血必净注射液、参附注射液、生脉注射液

（4）恢复期：肺脾气虚

临床表现：气短、倦怠乏力、纳差呕恶、痞满，大便无力，便溏不爽，舌淡胖，苔白腻。

推荐处方：法半夏 9g、陈皮 10g、党参 15g、炙黄芪 30g、茯苓 15g、藿香 10g、砂仁 6g（后下）

九、解除隔离和出院标准

体温恢复正常3天以上、呼吸道症状明显好转，肺部影像学显示炎症明显吸收，连续两次呼吸道病原核酸检测阴性（采样时间间隔至少1天），可解除隔离出院或根据病情转至相应科室治疗其他疾病。

十、转运原则

按照我委印发的《新型冠状病毒感染的肺炎病例转运工作方案》（试行）执行。

十一、医院感染控制

严格按照我委《医疗机构内新型冠状病毒感染预防与控制技术指南（第一版）》、《新型冠状病毒感染的肺炎防护中常见医用防护用品使用范围指引（试行）》的要求执行。

国家卫生健康委办公厅　　2020年2月4日印发

校对：杜青阳
