# Supplementary material for: Association of serum uric acid levels with COVID-19 severity
Source: BMC Endocr Disord. 2021 May 8;21:97. doi: 10.1186/s12902-021-00745-2 (PMC8106517; doi:10.1186/s12902-021-00745-2)
Supplement: Supplementary file 1 — Additional file 1: Table 1S Clinical classification of the COVID-19. [file 12902_2021_745_MOESM1_ESM.zip › Table 1S _ESM.docx]

**Table 1S：** Clinical classification of the COVID-19

| moderate group | | severe group | |
| --- | --- | --- | --- |
| Mild | Common | Severe | Critically severe |
| Mild clinical manifestation, no imaging performance. | Fever, respiratory symptoms, pneumonia performance on X-ray or CT. | Meet any of the following: 1. Respiratory distress, RR ≥ 30 beats/min.  2. Oxygen saturation ≤ 93% at rest state. 3. Arterial partial pressure of oxygen (PaO_2_)/Fraction of inspiration O_2_ (FiO_2_) ≤ 300 mmHg (1 mmHg = 0.133 kPa). | Meet any of the following: 1. Respiratory failure requiring mechanical ventilation. 2. Shock. 3. Combined with other organ failure, patients require ICU monitoring and treatment. |

**Note：**The COVID-19 patients were divided into the severe group (including severe and critically severe patients) and the moderate group (including mild and moderate patients) according to the fifth edition of China’s Diagnosis and Treatment Guidelines of COVID-19.
